# Beyond perfect synchrony: shared interpersonal rhythmic timing enhances self-other merging judgements

**DOI:** 10.1098/rsos.241501

**Published:** 2025-03-12

**Authors:** Dhwani P. Sadaphal, Christian R. Blum, Peter E. Keller, W. Tecumseh Fitch

**Affiliations:** ^1^ Department of Behavioral and Cognitive Biology, University of Vienna, Vienna, Austria; ^2^ Center for Music in the Brain, Department of Clinical Medicine, Aarhus University, Aarhus, Denmark; ^3^ The MARCS Institute for Brain, Behaviour and Development, Western Sydney University, Penrith South, Australia

**Keywords:** self-other merging, interpersonal rhythmic variation, synchrony, liking, perspective-taking, social bonding

## Abstract

Perfect synchrony is highly prosocial, yet interpersonal rhythms globally exhibit rich variation. In two online experiments, we tested the effect of varying interpersonal rhythms on self-other merging. First, we hypothesized that shared temporal features, acting as attentional frameworks to track and integrate self-other actions, would drive combined representations. Participants viewed and rated self-other pairs producing simple rhythms, polyrhythms and irregular rhythms, at three complexity levels. Merging was unsurprisingly highest for perfect synchrony and declined with other rhythmic ratios. Crucially, simpler polyrhythms were rated higher than irregular rhythms, supporting our tracking-and-integration hypothesis. Second, we tested whether interpersonal rhythmic variation specifically affected self-other merging versus aesthetic judgements, by collecting liking ratings for the identical stimuli. We hypothesized that liking would be driven by overall perceptual features versus interpersonal features. While ratings were unaffected by simple rhythms’ ratios, polyrhythms showed a sharp decrease, suggesting that social individuation inherent in polyrhythms additionally affected aesthetic judgements. The distinct liking pattern suggested that self-other merging judgements were specifically linked to the interpersonal nature of rhythmic variation, and not mere aesthetic preferences. Our data are consistent with the hypothesis that interpersonal rhythmic variation evolved to support prosocial bonds by signalling shared intentions and aiding clear self-other distinctions.

## 1. Introduction

Historically, human societies have relied on cooperation through interpersonal coordination to achieve shared goals [1]. Even common everyday activities such as walking together can display precise temporal coordination at both the individual and group level. Conversely, the social advantages of synchrony also lead to coordinated actions connoting shared feelings of cooperation and trust [2–7]. Group music-making poses challenges to interpersonal coordination, and achieving it successfully has positive social consequences [8]. Studies on the prosocial effects of synchrony demonstrate the bidirectional link between rhythmic interpersonal coordination and feelings of social bondedness [9–11]. In a pioneering functional magnetic resonance imaging (fMRI) study, Kokal *et al*. [12] showed that enhanced activity in social brain areas while drumming with synchronous versus asynchronous partners correlates with subsequent prosocial behaviour towards the synchronous partner. Social advantages of synchrony have been established consistently across a variety of participant groups, including adults [13–15], children [16,17] and even infants [18,19]. Patterns emerging from interacting rhythms may vary across several temporal features: their event structure may be isochronous (equally spaced) or heterochronous, their periods may be the same or related through numerical ratios, and the rhythms may be in-phase or out-of-phase [20]. Existing work on social bonding through rhythmic coordination encompasses several of these features. A seminal study linking synchrony to affiliation [13] assessed participants’ likeability ratings towards a partner whose isochronous tapping was manipulated to be either in-phase (i.e., synchronous) or phase-shifted relative to the participant’s tapping. Partner likeability was significantly higher for the synchronous over the phase-shifted tapping. Importantly, partner likeability increased only when the partner also tapped along synchronously, and not simply as a consequence of experiencing synchrony with a metronome in the presence of the partner. This bolstered the claim that it was the social nature of synchrony that drove increased feelings of affiliation. More research has since followed, showing that other stable forms of coordination, such as antiphase (i.e., alternating) coordination, sometimes prove just as socially effective as in-phase synchrony [21–24].

The psychological process of ‘self-other merging’ is one proposed mechanism underlying prosocial feelings evoked by interpersonal synchrony [11,13]. Typically, actions produced by the self and other entities are distinguished by mapping their distinct sensory consequences appropriately onto the self or the other. This mapping process can also incorporate others’ perspectives to simulate and predict the sensory outcomes of their actions. However, when individuals jointly produce a regular—and thus highly predictable—isochronous pulse, the brain forms an integrated representation of the self and the others’ actions due to their sensory alignment [8]. This merged representation, which implies aligned self and other perspectives, is possibly mediated by neural reward networks [12,25]. Stupacher *et al*. [15] devised an online perspective-taking paradigm to test within-participant measures of self-other merging and social bondedness across synchronous and asynchronous conditions. They asked participants to imagine themselves as being one of two stick figures on screen, walking together. The other figure walked either in-phase or out-of-phase with the self. Consistent with the self-other merging hypothesis, feelings of self-other merging, in addition to partner likeability and well-being, were found to be the highest when the two figures walked together synchronously.

However, recent investigations of more varied forms of interpersonal coordination suggest that bonding may not be limited to instances of perfect synchrony and other highly stable coordination [26–29]. For instance, Ravreby *et al*. [26] accounted for the role of novelty and complexity in addition to synchrony in a mirror game in which dyads were asked to move their hands in the most coordinated way possible. They found that participants liked each other the most when their movements were optimally novel and complex, despite this coming at a cost to the more predictable nature of complete synchrony. In extension, specific to musical rhythms, Savage *et al*. [30] propose an evolutionary function of rhythmic variation in their music and social bonding (MSB) hypothesis, arguing that variation maintains social engagement by balancing novelty against repetition. Indeed, it may be due to the diversity of communicative contexts that, considered globally, ensemble music contains rich and impressive rhythmic variation [31].

**Figure 1.**
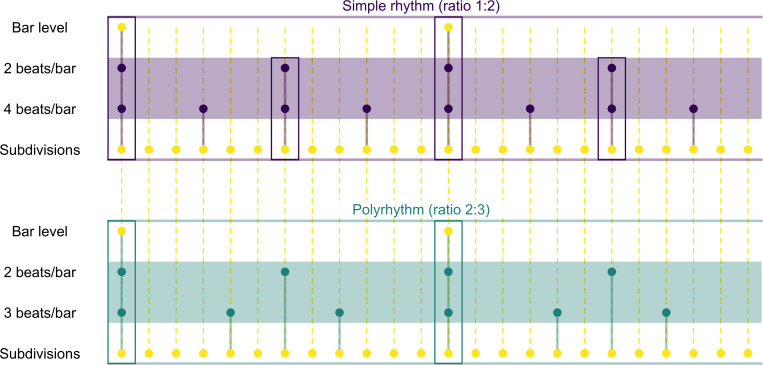
**A comparison of hierarchical timing (i.e., meter) in a 1 : 2 rhythm and a 2 : 3 polyrhythm.** The figure shows two bars of a simple two-stream 1 : 2 rhythm and a two-stream 2 : 3 polyrhythm. Yellow dots in the top row show the first beat of every bar. The two subsequent rows show the two streams contained in the simple rhythm (purple) and polyrhythm (green). Finally, yellow dots in the bottom row show temporal subdivisions common to both rhythms. The two streams in the simple 1 : 2 rhythm form regular (equal) intervals at the subdivision level, i.e., they form a periodic pattern that repeats twice per bar, overlapping at the first beat and then in the middle of the bar. In contrast, the two streams in the complex 2 : 3 polyrhythm group the underlying temporal subdivisions unequally, and therefore form a unique pattern repeated every bar but never within it, overlapping only at the first beat.

Ensemble music around the world generally requires complex interpersonal coordination that places high demands on humans’ cognitive faculties. A notable and globally prevalent case of such demanding coordination is that of music containing period-based temporal relationships [32]. In such music, multiple parts varying at multiple rates are spontaneously represented [33–35] within a structured cognitive hierarchy, known as ‘meter’ (figure 1). This hierarchy reflects the brain’s predictions about repeating rhythmic patterns and accents [36,37]. For instance, in a simple 2 beats per bar rhythm, a metrical pattern typically known as a ‘march’, the bar represents the broadest hierarchical level of the repeating rhythmic pattern, further subdivided by the occurrence of the beat at equal intervals, forming the next hierarchical level. Common metrical hierarchies are often unmistakable—a 3 beats per bar ‘waltz’ pattern would seldom be misjudged as a 4 beats per bar ‘salsa’. In several musical traditions and styles, rhythmic complexity is enhanced further by deliberately breaking this cohesive structure using polyrhythms (figure 1). These are rhythms that vary simultaneously along multiple distinct metrical hierarchies [37,38]. A distinct line of research on musical rhythms explores the role of rhythmic complexity on ‘groove’—the pleasurable desire to move to a beat. This research will be reviewed in §5, but note that it typically involves syncopated rhythms, whereas the rhythms we employ in this study are fully isochronous (unsyncopated) streams.

The current study addresses, in two experiments, the social effects of such period-based interpersonal rhythmic variation within the context of a metrical hierarchy.

### 1.1. Experiment 1

In Experiment 1, we tested the effect of systematically varying period-based relationships of rhythmic patterns between individuals on feelings of self-other merging in an online context. We hypothesized that a metrical hierarchy, in addition to temporal regularity of interpersonal rhythms, would act as a scheme for tracking and integrating self-other actions [8,39], leading to self-other merging (from here on, the ‘tracking-and-integration’ hypothesis). For this, we employed Stupacher *et al*.’s [15] perspective-taking paradigm. While this paradigm did not require participants to move explicitly, it captures psychological aspects of self-other integration, with the underlying assumption that individuals are generally able to project themselves onto behaving virtual figures and are sensitive to social connection in such contexts [3,10,15,21,27,40–43].

**Figure 2.**
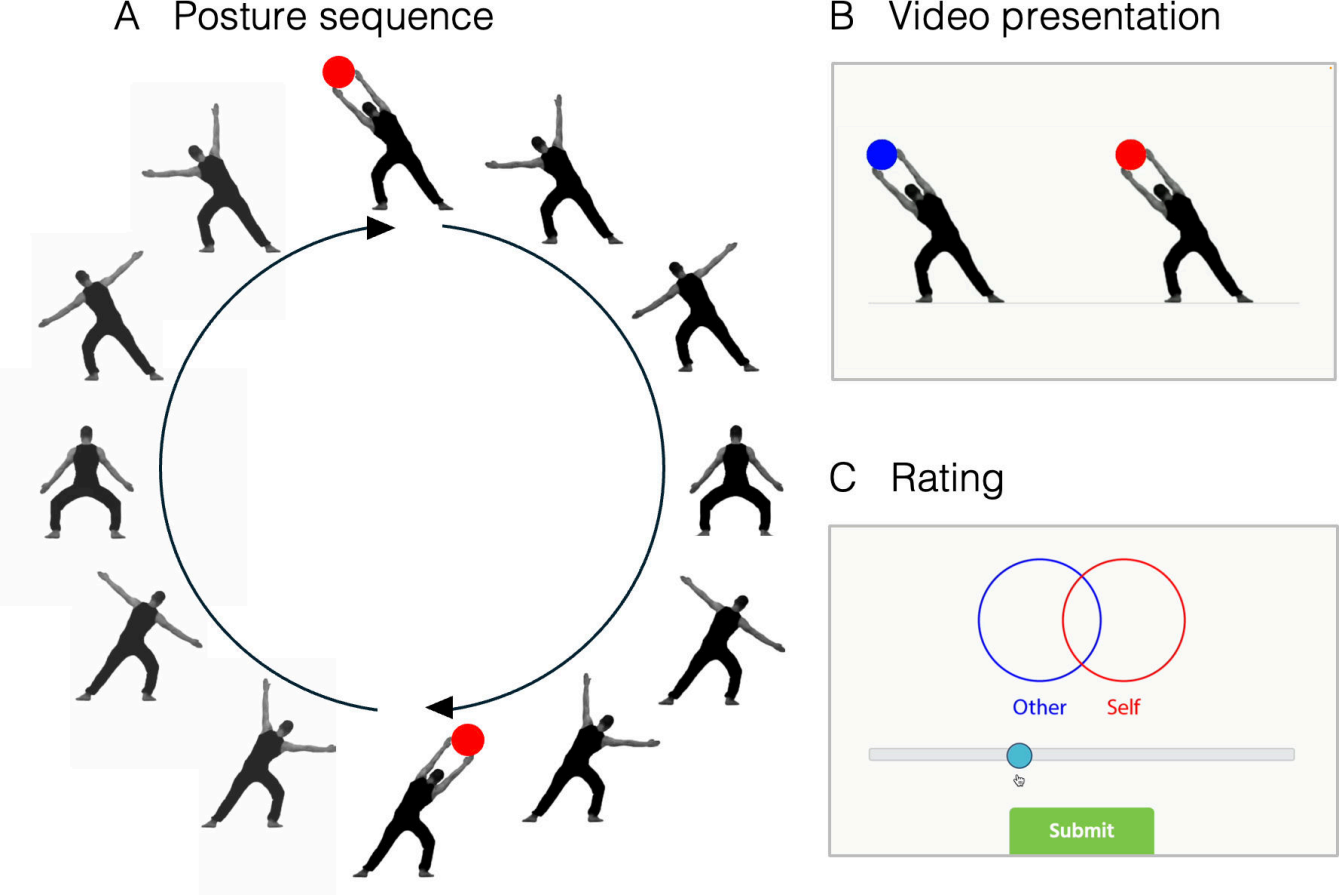
**Stimuli and main task.** (A) Ordered postures of a dancer used to construct the stimuli, (B) Final video stimuli presenting two dancers coordinating together to produce rhythms. Coloured circles appeared concurrently with the drum sounds. (C) The ratings for inclusion of the other in the self (IOS) were entered using a sliding 100-point scale that moved the two circles representing the ‘self’ and the ‘other’. The ratings for IOS were entered using a sliding 100-point scale that moved the two circles representing the ’self’ and the ’other’. Colours of the circles in the videos and the rating scale coincided with the position of the self on the screen (left/right) for each participant and also with the colours of the circles in the self and the other’s beats in the videos.

In the main task, participants were asked to imagine being one of two moving human figures presented in videos and rate their feeling of connectedness with the ‘other’ (figure 2B,C). We varied rhythmic coordination between the two figures using three two-stream ‘rhythm types’ (table 1): (i) ‘simple’ rhythms shared a metrical hierarchy at both the bar and subdivision levels, (ii) ‘polyrhythms’ shared a metrical hierarchy at the bar level but not at the subdivision level, and (iii) ‘irregular’ control rhythms had no meaningful metrical hierarchy. To control for rhythmic complexity at the individual level, individual streams in simple rhythms and polyrhythms were kept strictly isochronous. This isochrony differentiates the current study from much previous work on musical rhythm (which often uses syncopated rhythms) and aligns more closely with previous research in non-musical social bonding through music. Individual streams of irregular rhythms contained randomly generated inter-beat intervals. For these three rhythm types, we measured self-other merging as a representation of social bondedness on a modified continuous inclusion of the other in the self (IOS) scale [10,44] (figure 2C).

**Table 1.** The key manipulation of rhythm type and level of complexity

Rhythm type → Level of complexity ↓	Simple	Polyrhythm	Irregular
**Low**	1:1	2:3	Slow
**Medium**	1:2	3:4	Medium
**High**	1:3	4:5	Fast

Social bonding, in correspondence with the pleasurable feeling of wanting to move (termed ‘groove’ in the literature), also appears to have a ‘sweet spot’ for moderate levels of complexity, parametrized in several earlier studies using the syncopation index [45–48]. But unlike the aforementioned studies, we controlled for individual-level rhythmic complexity using easy-to-track isochronous pulse streams. Hence, we used the ratio between the relative inter-beat intervals of the two pulse streams as a proxy for the ‘level of complexity’ (table 1). This definition of complexity presupposes a predictive coding account of rhythm processing, which posits that the brain optimizes effort by reducing error in its predictions of future events [49,50]. For rhythms with period-based relationships, this predicts that more complex ratios would require more effort and are therefore less stable than simpler ratios. Empirical support for this account stems from studies of bimanual [51,52] and interpersonal [53] polyrhythmic coordination, and polyrhythm perception [54–56]. For irregular rhythms, where a ratio is not relevant, we assumed increasing complexity for slow, medium and fast tempi using arbitrary ranges of inter-beat intervals.

According to our *tracking-and-integration* hypothesis, we tested the significance of the key interaction between the independent variables rhythm type and the level of complexity to reflect the temporal features shared between individual streams. For simple rhythms and polyrhythms, this hypothesis suggests the following systematic effects of these shared temporal features on IOS, in comparison with the irregular control rhythms. Specifically, we predicted that simple rhythms—due to their shared subdivision and bar-level hierarchy—would receive the highest overall IOS ratings. Further, polyrhythms—being temporally regular despite having unequal subdivisions—would be rated higher than irregular rhythms [57] but lower than simple rhythms. To account for the absolute ratios between the two streams, we hypothesized two likely patterns for the effect of the level of complexity on IOS. First, if predictions are made based on the overall heterogeneous temporal pattern created by both streams, IOS ratings would show an inverted U-shaped relationship (a ‘sweet spot’) with increasing complexity [46]. Alternatively, if simultaneously attending to the self while integrating the other’s actions [12,58–61] proves too cognitively challenging, IOS ratings would show a decreasing linear relationship, i.e., a bias towards less complex rhythmic ratios.

In addition to the key manipulation of rhythm type and level of complexity, we controlled for participants’ rhythmic abilities by measuring their performance on a synchronization-continuation task [62]. Our analyses also considered participants’ empathic perspective-taking ability, which is known to factor into tasks involving rhythmic interpersonal coordination [9,10,63,64]. We measured participants’ trait empathy on the perspective-taking subscale of the brief interpersonal reactivity index [65] as a proxy for their ability to assume the ‘self’ role during the task [66].

### 1.2. Experiment 2

The MSB hypothesis [30] motivated us to further test the specificity of rhythmic variation to social contexts in a second experiment. Thus, in Experiment 2, we tested how our stimuli would be rated in a purely observational context with a new participant pool. We hypothesized that removing the requirement to track one pulse stream over another would encourage a holistic view of the two streams, potentially modulating aesthetic judgements of the rhythm, while simultaneously maintaining the interpersonal segregation of the two rhythmic streams among the moving figures. To this end, we utilized the same stimulus set, but instead simply asked participants to rate how much they liked the stimuli.

Aesthetic judgements of rhythm are known to rely on multiple perceptual features, such as beat grouping and diversity in temporal subdivisions [67,68]. Liking preferences also seem to show a relatively robust sweet spot for moderate complexity [46–48,69]. We therefore predicted that liking ratings would follow a separate pattern in Experiment 2 as compared with Experiment 1: polyrhythms would probably be perceived as more aesthetically pleasing because they prevent an easy inference of a metrical hierarchy by allowing two possible metrical interpretations. According to predictive coding theory, this is because the overall event structure of polyrhythms, containing the largest variety of subdivisions and grouping levels, encourages continuous predictive updates about likely temporal events. This predictive process becomes rewarding as predictions are successfully met with multiple repetitions of the polyrhythmic pattern. Following the same logic, we expected simple rhythms to be liked less due to their event structure fitting cohesively within one metrical hierarchy, leading to more comfortable yet less rewarding temporal predictions. Finally, we maintained our prediction that the irregular rhythms would get lower liking ratings than both simple rhythms and polyrhythms. This is because, while the irregular inter-beat intervals also require continuous predictive updates regarding temporal events, they are unlikely to be rewarding as they would practically prevent any successful predictions altogether.

## 2. Methods

### 2.1. Participants

We recruited a total of 50 participants (18 female, 32 male; age *M* = 32.18, s.d. = 9.63) in Experiment 1. Of these, we had to exclude three participants due to empty tapping recordings on the synchronization-continuation task, resulting in a total of 47 participants for Experiment 1. Experiment 2 also included 50 participants (31 female, 19 male; age *M* = 30.92, s.d. = 11.21) and none were excluded. Participants were recruited through the crowdsourcing service of the online experiment hosting platform Labvanced. Experiment 1 participants belonged to ten different countries (Germany, Greece, Hungary, Italy, Mexico, Poland, Portugal, South Africa, Spain and the United Kingdom). Experiment 2 participants belonged to 11 different countries (Belgium, Greece, Ireland, Italy, Japan, Mexico, Poland, Portugal, South Africa, Spain and the United Kingdom).

### 2.2. Stimuli

Stimuli were three sets of 12 videos each for the three interpersonal rhythm types: simple, polyrhythm and irregular, each with three different levels of complexity. Stimuli were created using custom Jupyter Notebook v. 4.2.2 (python v. 3.7) scripts using packages opencv-python v. 4.10.0, scipy v. 1.13.1, pyo v. 1.0.5, numpy v. 1.26.4 and moviepy v. 1.0.3. Videos are available in the electronic supplementary material.

#### 2.2.1. Simple and polyrhythm conditions

In the simple and polyrhythm conditions, videos were created by sequentially displaying still images of a dancer in 12 distinct postures (figure 2A), provided kindly by Guido Orgs. When ordered, concatenated and played back with interpolated frames at 60 frames per second, each image sequence showed one apparent movement trajectory: starting with hands fully extended to the left, bringing them downwards, fully extending them to the right and finally bringing them to the left again. Each full hand extension on either side coincided with the beat (along with a corresponding drum sound) in the isochronous pulse stream. A red or blue circle would also appear in the centre of the dancer’s fully extended hands to visually aid perception of the pulse (figure 2B).

In our stimuli, two such two dancers were displayed side by side, coordinating to produce the various rhythms (figure 2B). The final video was looped to show six complete bars of each rhythm. Participants were asked to take the perspective of one of these two dancers, designated the ‘self’ in the video. Participants were randomly assigned to groups with the ‘self’ on either the left or right side of the screen. (Note: our decision to use dissimilar video durations of the stimuli may be considered a confounding factor. However, we find it more pertinent to display an equal amount of information at bar level, i.e., six full bars for each rhythmic ratio, so that comparisons can be made based on the rhythm type and level of complexity of those rhythms. For irregular rhythms, the video durations of the three levels of complexity were matched with the range of variation in durations of the simple rhythm and polyrhythm videos.)

Our key manipulation involved different interpersonal rhythm ratios (table 1) as follows: the simple condition contained three absolute ratios at increasing levels of complexity (low, medium and high): 2 : 2, 2 : 4, 2 : 6 (discussed in the rest of the article in terms of the relative ratios 1 : 1, 1 : 2 and 1 : 3, respectively). The polyrhythm condition contained the ratios 2 : 3, 3 : 4 and 4 : 5, respectively. Similarly, tempi were calculated such that the faster dancer of the two, in all stimuli, moved at 90 b.p.m. Two sharp-onset rapid-decay drum sounds (i) ‘bell’ (*t*_onset_ = 0 s; *t*_decay_ = 0.5 s, *t*_fade_ = 0.15 s) and (ii) ‘knock’ (*t*_onset_ = 0 s; *t*_decay_ = 0.5 s; *t*_fade_ = 0.10 s) differentiated each distinct isochronous pulse stream. Drum sounds corresponding with each pulse stream and the speed of movement of the ‘self’ in relation to the ‘other’ (faster/ slower) were counterbalanced to produce four versions of each rhythm (videos in electronic supplementary material). Each of the resulting videos was assigned a unique stimulus ID. All participants were displayed all the video stimuli.

#### 2.2.2. Irregular condition

The irregular condition was created by randomly generating the display interval for each ordered dance posture, resulting in random inter-beat intervals. The inter-beat intervals varied randomly between three approximate windows: 83–333 ms (slow), 55–222 ms (medium) and 41–166 ms (fast) with step sizes of 16.5 ms, 11.2 ms and 8.34 ms, respectively. Drum sounds corresponding with each dancer were counterbalanced.

### 2.3. Procedure

#### 2.3.1. Experiment 1

Before beginning the experiment, participants were asked to wear headphones, provide informed consent and report relevant demographic and musical background information. Participants proceeded with self-adjusting the playback volume to set it to a comfortable listening level as they heard an isochronous pulse with the ‘bell’ sound. This was followed by three trials of a synchronization-continuation task [62] in which they had to synchronize with a 90 b.p.m. pulse stream. They were instructed to start tapping 2.6 s into the playback. The playback was stopped at 12 s, while they continued tapping, trying to maintain the same tempo for a further 12 s.

In the main session, participants were instructed to imagine themselves in the role of ‘the self’ producing a rhythm with an unknown ‘other’ in a video that was displayed to them upon self-initiating a trial by clicking a button. All 36 video stimuli were displayed to each participant in pseudorandom order, while ensuring that no version of the same period-based ratio was repeated in consecutive trials. The end of every trial displayed the question, ‘How connected do you feel to the other person after this activity?’. The response was measured using a modified inclusion of the other in the self [10,44] i.e., ‘IOS’ scale. This rating was indicated by dragging a slider on a scale ranging from 0 to 100 with increments of 1 (figure 2C). Each incremental movement of the slider controlled a Venn-diagram display of two circles representing the self and the other. The position of the circles ranged from almost totally overlapping to the extreme left to non-overlapping on the extreme right. The number on the scale was not displayed to the participant. Following the main session, participants completed the Brief Interpersonal Reactivity Index [65] i.e., ‘B-IRI’.

The main session was preceded by a practice session in which participants viewed the same instructions and gave IOS ratings for five videos—encompassing all rhythm types and extremes of complexity—differing in tempi from the main session stimuli. Practice trials were not analysed.

#### 2.3.2. Experiment 2

Experiment 2 was identical to Experiment 1, excepting the omission of the B-IRI questionnaire and the question in the main and practice sessions being changed to ‘How much did you like the video?’. In this experiment, participants’ liking ratings between 0 and 100 for each video were recorded by simply dragging the slider.

### 2.4. Audio processing

Data from the synchronization-continuation task in both experiments were processed on Python using a custom Jupyter Notebook v. 4.2.2 (python v. 3.7) script utilizing packages scipy v. 1.13.1 and librosa v. 0.10.2. Each participant performed three synchronization-continuation trials. For each of the three trials, recordings were bandpass filtered, followed by envelope detection and normalization of amplitude. Events crossing 40% of the maximum onset strength in each recording were detected as tap onsets. Asynchronies were then calculated as the absolute time difference between detected onsets and the onsets of the 90 b.p.m. target pulse, for a 10 s chunk of the recording after playback was stopped. A single value capturing the variability of tap onset asynchronies (from here on, ‘tapping asynchrony’) for each participant was calculated by taking the mean of the standard deviation of tap onset asynchronies in all three synchronization-continuation trials.

## 3. Statistics

### 3.1. Generalized linear mixed model analysis (both experiments)

See table 2 for package and version details for functions mentioned in §2. Data from both experiments were analysed on R version 4.3.2 [70] using GLMMs [71,72] with the function glmmTMB [73]. For both experiments, we compared a full model, reflecting the alternative hypothesis, with a null model, reflecting the null hypothesis [74]. The reasoning behind this was to test all test predictors together at once in a single comparison to avoid cryptic multiple testing [75]. The null model was identical to the full model except that it lacked the main effects and interaction of our key test predictors ‘rhythm type’ and ‘level of complexity’. If the full model proved to be a better fit than the null model, we proceeded with the individual fixed effects of the test predictors in the full model using likelihood ratio tests [76] with the drop1 function (test argument = ‘Chisq’). If the effect of the interaction term was significant, we proceeded with a post hoc Tukey test with *p*-value adjustment to compare pairwise contrasts in estimated marginal means of levels in the interaction of key test predictors using the function emmeans [77]. The full model in both experiments was defined with a beta error distribution with a logit link function. The fixed and random effects structure for Experiment 1 and 2 models and model diagnostics are discussed in detail separately in the following sections.

**Table 2.** Summary of functions and R package versions used for statistical analyses.

Purpose	Function	Arguments	Package	Version
Generalized linear mixed model definition	glmmTMB	family = beta_family(link = ‘logit’)	glmmTMB	1.1.8
Maximal random effects structure determination	fe.re.tab	-	Mundry, 2023	-
Parametric bootstrap, 95% confidence intervals	boot.glmmtmb	use = c('rhythm.type', 'level.of.complexity')	Mundry, p.c.	-
Overdispersion test	overdisp.test	-	Mundry, p.c.	-
Collinearity test using variance inflation factors	vif	-	car	3.1.2
Model definition for collinearity test	lmer	-	lme4	1.35.3
Null to full model comparison	anova	test = 'Chisq'	base	4.3.2
Individual effects of predictors	drop1	test = 'Chisq'	base	4.3.2
Effect size estimation	r2	-	performance	0.11.0
Effect size estimation	r.squaredGLMM	-	MuMIn	1.47.5
Post hoc pairwise contrasts	emmeans	-	emmeans	1.10.2

To keep type I error rate at the nominal level of 0.05, we defined a maximal random effects structure with all theoretically identifiable random slopes [74,76], using the function fe.re.tab [78]. Initially, we also included correlations between random intercepts and slopes; however, this resulted in the model being too complex as indicated by the maximum log-likelihood of the model being undefined [79]. We therefore removed them from the full model. The 95% confidence intervals were obtained using the custom function boot.glmmtmb (Mundry 2024, personal communication), using 1000 parametric bootstrap and bootstrapping over the random effects (argument ‘use’ set to include the key test predictors). Finally, to allow comparability across other studies, we cite effect sizes from two different R packages: (i) using the function r2 [80] and (ii) using the function r.squaredGLMM [81].

### 3.2. Experiment 1: Inclusion of the other in the self

We modelled the dependent response variable ‘IOS rating’ as a function of independent key test predictors ‘rhythm type’ interacting with ‘level of complexity’. Additional independent control predictors—added as fixed effects—included ‘empathic perspective-taking’ (mean of the four items of the perspective-taking subscale of the B-IRI), ‘tapping asynchrony’ as described above, the ‘side of the self’ (left/right), ‘gender’, the ‘drum sound’ corresponding with the self and ‘trial number’. ‘Participant ID’ was included as a random effect with random slopes for rhythm type, level of complexity, their interaction and drum sound. Additionally, ‘stimulus ID’ was included as a random effect with random slopes for empathic perspective-taking, tapping asynchrony, side of self, gender, drum sound and trial number. All covariates (empathic perspective-taking, tapping asynchrony and trial number) were z-transformed, i.e., scaled to a mean of zero and a standard deviation of 1, to ease model convergence. All factors (rhythm type, level of complexity, side of self, gender and drum sound) were dummy coded for ease of interpretation and centred to an approximate mean of zero.

We performed diagnostics to check model assumptions. A test of overdispersion using the custom function overdisp.test (Mundry 2024, personal communication) showed the model to be mildly underdispersed (dispersion parameter = 0.89), leading to somewhat conservative model estimates. Underdispersion leads to more conservative models and increased risk of type II errors. It is therefore considered less of an issue than overdispersion, which leads to more liberal models and increased risk of type I errors. We assessed variance inflation factors (VIFs) to test for collinearity of predictors using the function vif [82] on a lmer linear model [83]. This model was identical to the full model, but lacking the interaction term (still including their main effects), and lacking all random effects [84–86]. This yielded squared generalized VIFs taken to the power of 1/(2 × the respective degrees of freedom) approximately equal to 1 (max(sq(GVIF^(1/(2 × d.f.))) = 1.1) within acceptable limits [87]. A visual inspection of plots of the best linear unbiased predictors (BLUPs) showed approximately normal distributions [71,88] within acceptable ranges (max. range = 4). We assessed model stability by excluding levels of random effects one at a time and comparing the estimates to those of the full model [89]. This revealed rather small ranges for all model components excepting moderate ranges for three random slopes within participant ID. This revealed our model to be highly stable.

Using the function r.squaredGLMM [81], we obtained R2m = 0.41 and R2c = 0.74. Using the function r2 [80], we obtained a marginal R squared (R2m) = 0.612. The package could not handle the random effects, resulting in an undefined conditional R squared (R2c = NaN).

### 3.3. Experiment 2: Liking

The full model for Experiment 2 was identical to the structure of the full model for Experiment 1 with the dependent response variable in this case being the ‘liking rating’ and independent key interaction of the test predictors rhythm type and level of complexity. Relevant independent control predictors included tapping asynchrony, gender and trial number, which were all included as fixed effects. Random effects included participant ID with random slopes for rhythm type interacting with level of complexity and stimulus ID with random slopes for tapping asynchrony, gender and trial number. Covariates (tapping asynchrony and trial number) were z-transformed, and factors (rhythm type, level of complexity and gender) were dummy coded and centred.

Diagnostic tests revealed the model to be somewhat underdispersed (dispersion parameter = 0.75). VIFs (max(sq(GVIF^(1/(2*d.f.))) = 1.06) were within acceptable limits [84–86]. Plots of BLUPs showed approximately normal distributions within acceptable ranges [71,88] (less than 4), excepting relatively wide ranges for high complexity irregular rhythms (range = 7) and 3 : 4 (range = 6) and 4 : 5 (range = 6) polyrhythms. Despite the wider ranges of BLUPs, the test of model stability [89] showed the model to be relatively stable, excepting the same three random slopes within participant ID reported for the BLUPs highlighted above. Given the design of Experiment 2, these ranges can be explained by the anticipated inter-individual variability in preferences for different period-based rhythmic ratios that are rather cognitively demanding to perceive.

The function r.squaredGLMM [81] resulted in effect sizes R2m = 0.29 and R2c = 0.69. Effect sizes using the function r2 [80] were R2m = 0.478 and R2c = NaN.

## 4. Results

### 4.1. Experiment 1

**Figure 3.**
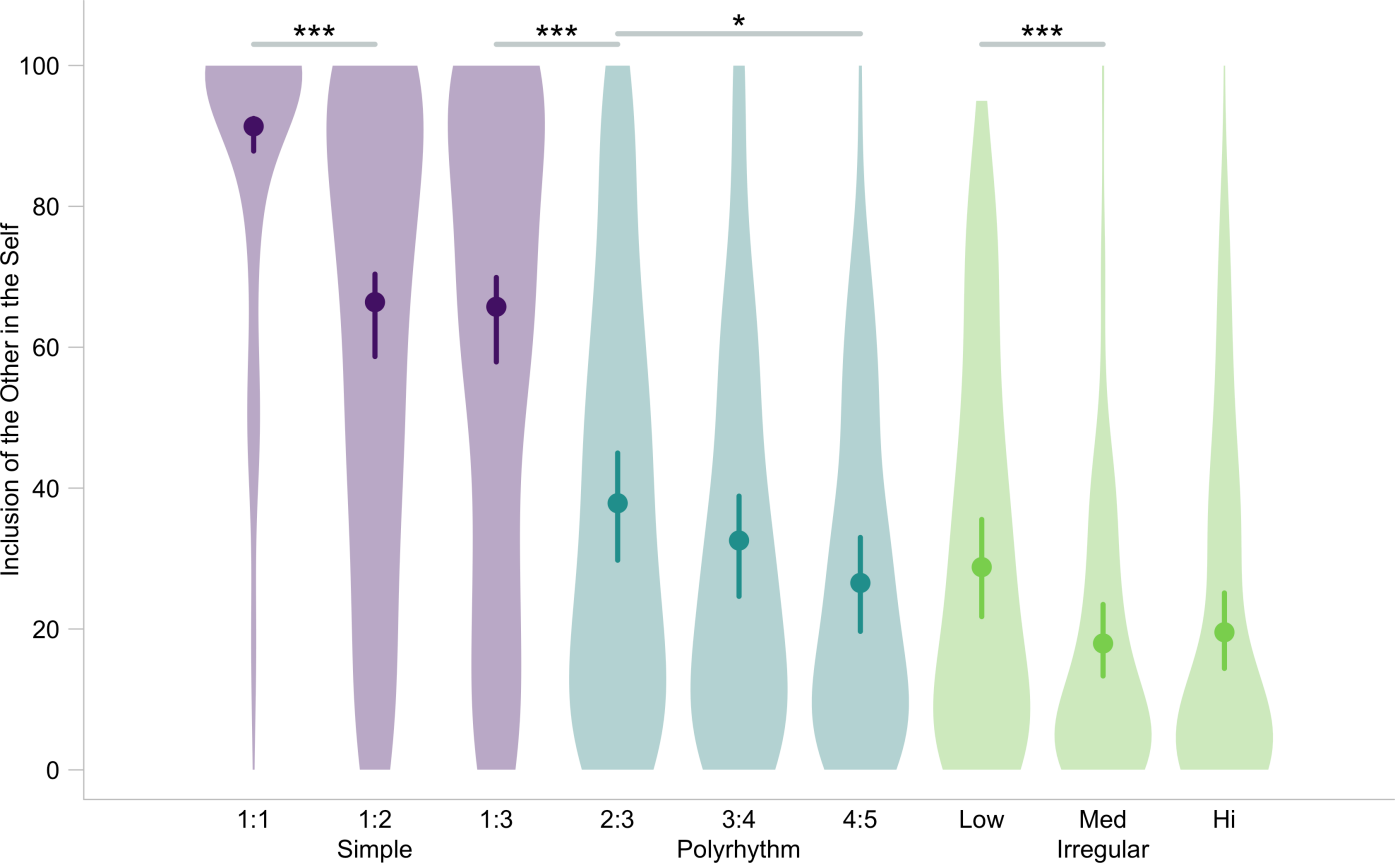
**IOS ratings across rhythm type and levels of complexity with confidence intervals and raw data.** Bootstrapped model fitted estimates of IOS ratings (circles). Segments show 95% confidence intervals for the interaction of key test predictors rhythm type (simple, polyrhythm and irregular) and level of complexity (low, medium and high) on the x-axis, conditional on the effects of all other covariates and factors being zero. x-axis labels for simple rhythms and polyrhythms depict the rhythm ratios. Shaded violin plots depict kernel density of raw IOS ratings across each rhythm type and level of complexity. Significance codes: * < 0.05, *** < 0.001

Figure 3 shows a plot of inclusion of the other in the self (IOS) ratings against our key test predictors ‘rhythm type’ (simple, polyrhythm and irregular) and ‘level of complexity’ (low, medium and high). We first tested our *tracking-and-integration* hypothesis by comparing a full GLMM representing the hypothesis against a null GLMM [74] (details in §2) representing the null hypothesis. The full model contained the main effects and interaction between our key predictors and response variable IOS. The null model lacked the test predictors but was otherwise identical to the full model. The key test interaction was significant, i.e., a Chi-squared test indicated that the full model explained the data much better than the null model (*χ*^2^ = 154, d.f. = 33, *p* < 0.001). Table 3 shows the summary of the full model.

**Table 3.** Model summary in link space for response variable IOS and the interaction of key test predictors rhythm type and level of complexity.

Explanatory variables	Estimate	Standard error	*z* value	*p*-value	
(Intercept)	2.12	0.23	9.28	< 0.001	^c^
Rhythm type polyrhythm^d^	−2.85	0.19	−15.03	< 0.001	^c^
Rhythm type irregular^d^	−3.26	0.21	−15.27	< 0.001	^c^
Level of complexity medium^d^	−1.68	0.12	−14.09	< 0.001	^c^
Level of complexity high^d^	−1.71	0.12	−13.76	< 0.001	^c^
Gender male^d^	0.51	0.21	2.45	0.02	^b^
Drum sound knock^d^	−0.15	0.20	−0.75	0.75	-
Self on screen right side^d^	0.02	0.05	0.32	0.45	-
Empathic perspective-taking^e^	0.19	0.10	1.79	0.08	^a^
Tapping asynchrony^e^	−0.18	0.10	−1.76	0.08	^a^
Trial number^e^	0.12	0.03	4.42	< 0.001	^c^
R. type polyrhythm: Level medium (3 : 4)	1.44	0.17	8.62	< 0.001	^c^
R. type irregular: Level medium	1.06	0.15	6.95	< 0.001	^c^
R. type polyrhythm: Level high (4 : 5)	1.18	0.19	6.16	< 0.001	^c^
R. type irregular: Level high	1.20	0.16	7.40	< 0.001	^c^

^a^ <0.1

^b^ < 0.05

^c^ < 0.001

^d^ Reference levels: ‘simple’ for rhythm type, ‘low’ for level of complexity, ‘female’ for gender, ‘bell’ for drum sound and ‘left side’ for self on screen.

^e^ z-transformed to an approximate mean of zero and s.d. of 1.

We followed this with likelihood ratio tests of the interaction of the key test predictors [76] (details in §2). Consistent with the *tracking-and-integration* hypothesis, the interaction of rhythm type and level of complexity had a significant effect (*p* < 0.001) on IOS, permitting us to continue with post hoc testing. We attribute the significant effect of control predictor trial number (*p* < 0.001; electronic supplementary material, figure S1b) to possible learning effects for specific rhythm ratios. Gender also had an effect (*p* < 0.05; electronic supplementary material, figure S1a) with lower ratings given by female participants. We speculate that this could be due to the unambiguously masculine appearance of the figures in our videos. Borderline significant predictors empathic perspective-taking (increasing trend) and tapping asynchrony (decreasing trend) are depicted in electronic supplementary material, figure S1c,d.

A post hoc comparison of pairwise estimated marginal means (details in §2; all contrasts in electronic supplementary material, table S2) showed that rhythm types were viewed as significantly different between the simple and polyrhythm categories (post hoc 1 : 3 versus 2 : 3: *e* = 1.7, s.e. = 0.20, *p* < 0.001). But the boundary between polyrhythms and irregular rhythms was blurred at the threshold (post hoc 4 : 5 versus irregular-slow: *e* = 0.1, s.e. = 0.26, *p* = 1). The lack of a difference between the 4 : 5 and slow irregular rhythm is not surprising, given that the 4 : 5 polyrhythm is relatively complex, and may thus evade regularity-detection, making it too difficult for most participants to track and integrate self-other actions. Bootstrapped IOS ratings (figure 3; details in §2) show these post hoc trends against levels of the key interaction.

Regarding the relationship between IOS and level of complexity, we found (i) neither an inverted-U nor a linear trend for simple rhythms, (ii) a linear trend for polyrhythms, and (iii) again neither an inverted-U nor a linear trend for irregular rhythms. Additionally, there was no overall inverted-U trend among the rhythm types. The 1 : 1 rhythm showed disparately high IOS in comparison with all other rhythms, differing significantly from even the simple 1 : 2 (post hoc: *e* = 1.7, s.e. = 0.12, *p* < 0.001) and 1 : 3 (post hoc: *e* = 1.7, s.e. = 0.12, *p* < 0.001) rhythms. While IOS ratings for polyrhythms were overall lower than all simple rhythms, a significant difference between the low and high complexity extremes (post hoc 2 : 3 versus 4 : 5: *e* = 0.5, s.e. = 0.14, *p* < 0.05) supported the prediction that the degree of regularity has an effect on IOS in polyrhythms. Unexpectedly, the slow irregular rhythm was significantly different from the other two irregular rhythms (post hoc slow versus medium: *e* = 0.6, s.e. = 0.12, *p* < 0.001; post hoc slow versus fast: *e* = 0.5, s.e. = 0.12, *p* < 0.001). This may be akin to a perceptual ‘resetting’ phenomenon, possibly spurred by the slowness of the stimuli and further compounded by the perspective-taking demand of the task, that results in an illusion of regularity when the brain processes irregular sequences [90–92]. In general, the absence of an inverted-U relationship between IOS and rhythm type opposes the idea of a complexity ‘sweet-spot’, driven simply by temporal heterogeneity, in an interpersonal context. But this is consistent with our alternative hypothesis about simpler ratios being relatively easier to track and integrate, leading to higher IOS.

Random effects in the model were mostly unremarkable, with the exception of random slopes of the 3 : 4 and 4 : 5 polyrhythms and the fast irregular rhythm within participant ID (electronic supplementary material, table S1, bold s.d. estimates). This suggests that individual participants varied considerably in their IOS ratings for the 3 : 4 and 4 : 5 polyrhythms and the fast irregular rhythm. The high variability in IOS for the aforementioned polyrhythms may be associated with difficulty tracking and integrating the streams, while variability in IOS ratings for the irregular rhythms may have been due to participants giving IOS ratings arbitrarily.

### 4.2. Experiment 2

**Figure 4.**
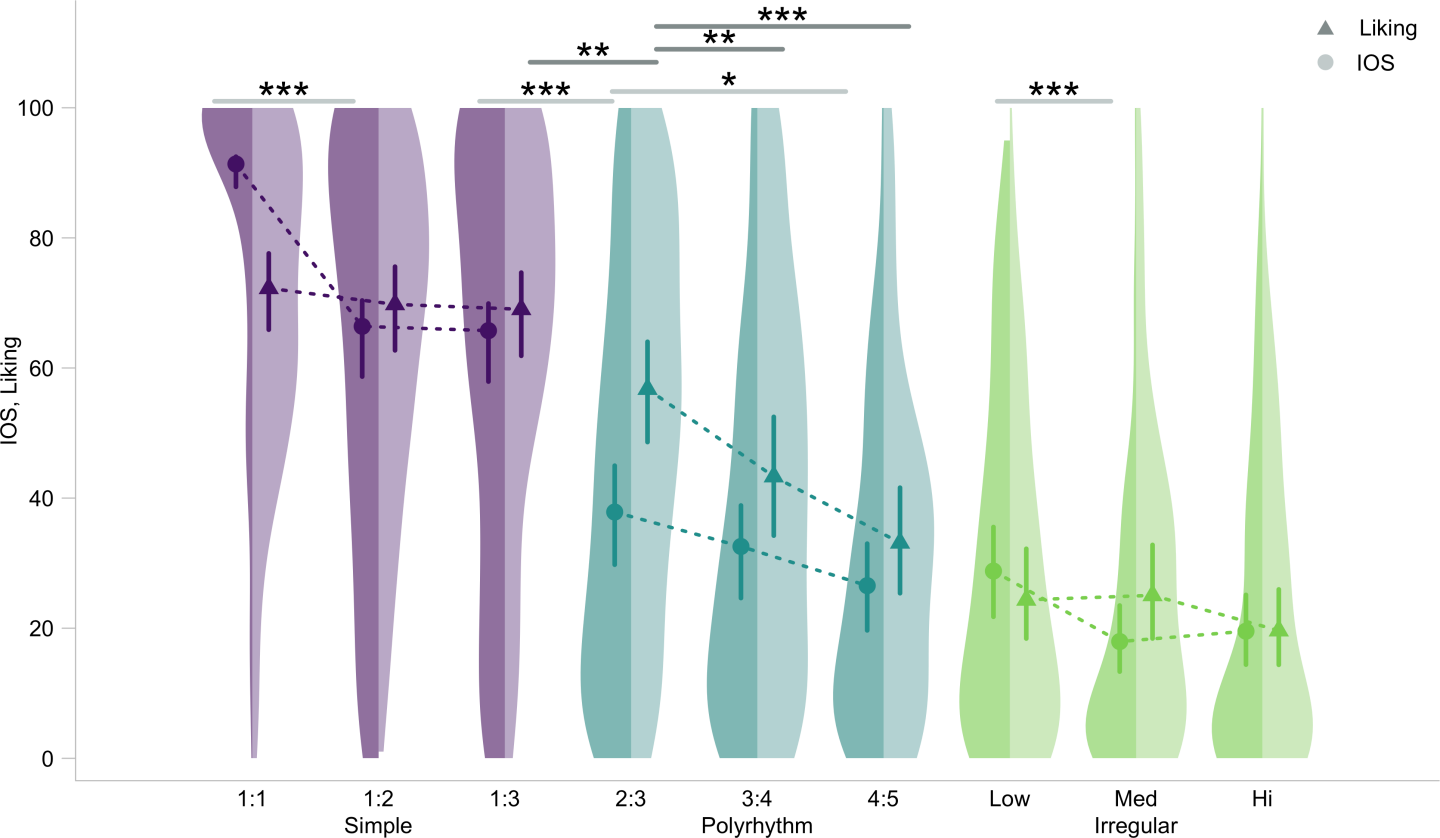
**IOS and liking ratings from both experiments with confidence intervals and raw data.** Bootstrapped model fitted estimates of liking ratings (triangles) from Experiment 2 and IOS) ratings (circles) from Experiment 1. Segments show 95% confidence intervals for the interaction of test predictors rhythm type (simple, polyrhythm and irregular) and level of complexity (low, medium and high) on the x-axis, conditional on the effects of all other covariates and factors being zero. x-axis labels for simple rhythms and polyrhythms depict the rhythm ratios increasing in complexity. Light shaded half-violin plots on the right depict kernel density of raw liking ratings from Experiment 2, and dark shaded half-violin plots on the left depict kernel density of raw IOS ratings (from Experiment 1) across each rhythm type and level of complexity. Dotted lines join the estimates from each experiment within each rhythm type. Significance codes (light grey for Experiment 1, dark grey for Experiment 2): * < 0.05, ** < 0.01, *** < 0.001

Figure 4 presents Experiment 2 liking ratings and together with Experiment 1 IOS ratings across levels of the key interaction of rhythm type and level of complexity, using identical video stimuli in both experiments with separate participant pools. Here we tested our hypothesis that aesthetic ratings would depend on overall rhythmic features in accordance with predictive coding theory, using a full-null model comparison [76] (details in §2). The full model contained the main effects and interaction between our key predictors ‘rhythm type’ and ‘level of complexity’ and response variable liking. The null model was identical to the full model but lacked the key test predictors. The Chi-squared test indicated that the inclusion of the key test predictors significantly improved model fit (*χ*2 = 154, d.f. = 33, *p* < 0.001). Table 4 summarizes the full model.

**Table 4.** Model summary in link space for response variable Liking and the interaction of key test predictors rhythm type and level of complexity.

Explanatory variables	Estimate	Standard error	*z* value	*p*-value	
(Intercept)	0.98	0.17	5.65	< 0.001	^b^
Rhythm type polyrhythm^c^	−0.68	0.17	−4.00	< 0.001	^b^
Rhythm type irregular^c^	−2.09	0.20	−10.46	< 0.001	^b^
Level of complexity medium^c^	−0.12	0.13	−0.93	0.35	-
Level of complexity high	−0.16	0.12	−1.27	0.20	-
Gender male^c^	−0.05	0.23	−0.21	0.83	-
Tapping asynchrony^d^	0.11	0.11	0.98	0.33	-
Trial number^d^	0.14	0.03	5.51	< 0.001	^b^
R. type polyrhythm: Level medium (3 : 4)	−0.42	0.23	−1.84	0.07	^a^
R. type irregular: Level medium	0.16	0.14	1.12	0.26	-
R. type polyrhythm: Level high (4 : 5)	−0.82	0.23	−3.54	< 0.001	^b^
R. type irregular: Level high	−0.12	0.15	−0.80	0.42	-

^a^ < 0.1

^b^ < 0.001

^c^ Reference levels: ‘simple’ for rhythm type, ‘low’ for level of complexity and ‘female’ for gender.

^d^ *z*-transformed to an approximate mean of zero and s.d. of 1.

Likelihood ratio tests of the interaction of the key test predictors [76] (details in §2) were consistent with the hypothesis that the interaction of rhythm type and level of complexity had a significant effect on liking (*p* < 0.05). We therefore continued with post hoc testing. The control predictor trial number was once again significant (*p* < 0.001, electronic supplementary material, figure S2). However, an effect of gender was notably absent here, implying that the social nature of the perspective-taking task may have evoked gender-based dynamics, affecting IOS ratings in Experiment 1.

Contrary to our predictions, post hoc contrasts showed that simple rhythms, and not polyrhythms, received the highest ratings overall (details in §2; all contrasts in electronic supplementary material, table S4, bootstrapped results in figure 4). In fact, a significant drop in liking only occurred at the transition between simple rhythms and polyrhythms (post hoc 1 : 3 versus 2 : 3: *e* = 0.53, s.d. = 0.16, *p* < 0.05; figure 4). In other words, the notable advantage of 1 : 1 synchrony, seen previously in Experiment 1, was no longer relevant in the observational context (post hoc 1 : 1 versus 1 : 2: *e* = 0.12, s.d. = 0.13, *p* = 0.99; post hoc 1 : 1 versus 1 : 3: *e* = 0.16, s.d. = 0.54, *p* = 0.94). Moreover, the 4 : 5 polyrhythm did not differ significantly from the slow irregular rhythm (post hoc: *e* = 0.43, s.d. = 0.25, *p* = 0.71). Interestingly, we found a steeper linear trend for polyrhythms (post hoc 2 : 3 versus 4 : 5: *e* = 0.97, s.e. = 0.16, *p* < 0.001; figure 4). These patterns are again inconsistent with the prediction of the moderate complexity ‘sweet spot’, but note that our definition of complexity differs from earlier studies. Nevertheless, caveated by the fact that the simple 2 : 3 polyrhythm is already fairly complex, the obtained patterns support the prediction about aesthetic judgements for polyrhythms being in accordance with predictive coding theory.

Random effects in Experiment 2 contributed relatively little to the model’s estimates of liking (electronic supplementary material, table S3). The clearest contributions were again seen for random slopes of the 3 : 4 and 4 : 5 polyrhythms and the fast irregular rhythm within participant ID (electronic supplementary material, table S3; bold s.d. estimates). Particularly, the variability in ratings for irregular rhythms remained relatively consistent across the two experiments, but liking ratings for the different polyrhythms varied even more widely in Experiment 2 than IOS ratings in Experiment 1.

## 5. Discussion

This study investigated the effects of variation in interpersonal rhythms with two online experiments. The first experiment employed a perspective-taking paradigm to test the effects of varying ratios of interpersonal period-based rhythms on feelings of self-other merging, measured by IOS ratings. The second experiment tested the specificity of the effects of interpersonal rhythmic variation to self-other merging, by obtaining simple liking ratings for the same stimulus set. In Experiment 1, we found that the interaction of the type of rhythmic variation (simple, polyrhythm or irregular) and the level of complexity of the rhythm (low, medium and high) had a significant effect on inclusion of the other in the self (IOS) ratings. Specifically, the 1 : 1 rhythm received unsurprising ceiling IOS ratings, followed by a linear decreasing trend as ratios increased in complexity. Nonetheless, several complex ratios were rated significantly higher than the irregular control rhythms. This was consistent with our hypothesis that interpersonal rhythmic variation within levels of a metrical hierarchy, possibly mediated by neural processes tracking and integrating self-other actions, modulates feelings of self-other merging.

In Experiment 2, we found that liking ratings when observing the same interpersonal rhythmic variation were also affected by the rhythm type and level of complexity. However, they showed a distinct pattern: simple rhythms were liked overall more than others, but the complexity of the period-based ratios of those rhythms had no significant effect. By contrast, we found a strong effect of period-based ratios for polyrhythms. This supported our hypothesis that an observational context modulates aesthetic judgements of the overall rhythm, leading to a separate pattern for liking in comparison with self-other merging. Interestingly, the results for liking and IOS may not be fully independent, as we found that mapping the two distinct rhythmic streams onto the two agents in the stimuli may have played an additional role decreasing liking ratings for the more complex polyrhythms. Thus, even though liking ratings did not sufficiently explain IOS, within the broader context of the study, the divergent patterns obtained for liking versus IOS ratings imply that IOS judgements were driven specifically by the social nature of interpersonal rhythms and not simply by aesthetic judgements about the stimuli. Broadly, our findings are relevant to (i) the role of interpersonal rhythmic variation in synchrony’s prosocial consequences, (ii) the perceptual features and cognitive representations driving aesthetic judgements regarding interpersonal rhythms, and have further implications regarding (iii) the mechanisms underlying the social bonding effects of complex rhythmic coordination.

We will initially underline some limitations of our study, arising mainly due to the online setting. An online task may not be ideal to investigate social bonding feelings. Ecologically, rhythmic variation is often produced during evocative (e.g. ritualistic, celebratory) social occasions where individuals’ willingness to coordinate is highly primed and/or practised. Prosocial effects of coordination are sometimes overridden by simple cues, e.g. tardiness [93] and broader social narratives, e.g. religiosity [94]. Thus, judgements made in an online setting may not generalize across real-life contexts. Furthermore, given our diverse sample, factors such as participants’ cultural backgrounds could eclipse important individual differences and add unexplained heterogeneity in the data. Cultural backgrounds could specifically influence mediating variables of rhythm processing and appreciation [64,95] and possibly due to variability in the interpretation of the IOS scale. Notably, studies using the IOS scale tap into related yet distinct phenomena associated with social closeness [10,15,46,58,64,96,97] and joint agency [27,29]. This makes participants’ judgements particularly susceptible to nuances in the phrasing of the question. Specific to our study, repeatedly evaluating one’s connectedness towards an animation of the same human figure may not be the most naturalistic exercise. Online settings may also obscure certain moderating strategies which might be at play when forming self-other representations in rhythmic tasks. For instance, genuine coordination in a laboratory setting reveals emergent patterns such as leading-following to achieve success, including polyrhythmic patterns [27,53]. Lastly, further studies are needed to explain the idiosyncratic variation in ratings towards relatively complex rhythmic ratios observed in our data. Nonetheless, our rationale behind using an online paradigm was that it would allow us to parametrically vary interpersonal rhythmic relationships, which generally exceed the average participant’s skill level, while circumventing the need for participants to produce it. An online setting further controls for other (e.g. olfactory, group-based) confounds, and crucially permits a more diverse multinational—although still English-speaking and technology-literate—participant pool (see §2 for countries of origin). These caveats should be kept in mind during the following discussion of results.

### 5.1. Shared timing in rhythmic variation modulates self-other merging

Our prediction regarding the interaction of key predictors rhythm type and level of complexity on IOS ratings was supported by the full-null model comparison and a subsequent individual likelihood ratio test. In the perspective-taking task, the 1 : 1 rhythm received overwhelmingly superior IOS ratings; on average approximately 85 points higher than the other two simple 1 : 2 and 1 : 3 ratios. This supports findings from previous literature showing that 1 : 1 correspondence is significantly more effective in producing feelings of self-other merging over phase-shifted or asynchronous coordination [12–15,18,21,58]. The resulting merged representation of the self and the other consequently encourages prosocial behaviour towards synchronous others.

We found that the average IOS ratings for the 2 : 3 and 3 : 4 polyrhythms were significantly higher by approximately 70 points than those of the medium and fast irregular rhythms. This finding is crucial to the *tracking-and-integration* hypothesis because it suggests that the bar-level regularity of polyrhythms, which was absent from the irregular control condition, led to stronger feelings of self-other merging. Additionally, a significant increase of approximately 25 points in IOS ratings for the 1 : 2 and 1 : 3 rhythms in comparison with the 2 : 3 polyrhythm suggests that the subdivision-level regularity further aided self-other merging. Taken together, rhythms with unequal period-based ratios showed a decreasing trend of IOS with increasing complexity. This is compatible with our reasoning that increasing the challenge of tracking and integrating self-other actions biases self-other merging judgements towards simpler interpersonal rhythmic ratios.

Overall, our findings do not only replicate the robust prosocial effect of 1 : 1 synchrony, but also align with recent studies which suggest that more complex forms of interpersonal coordination are also conducive to prosocial outcomes [21,23,27,53], particularly in scenarios where perfect synchrony fails to induce the most potent prosocial effects [26,28,29,94]. Hence, the study augments the current understanding of the prosocial effects of rhythmic coordination by showing that the degree to which temporal features of individual rhythms are shared along a metrical hierarchy systematically modulates feelings of self-other merging. The findings are also consistent with the MSB hypothesis [30] that rhythmic variation, in addition to simple synchrony, serves an evolutionary prosocial function.

### 5.2. Interpersonal rhythmic variation differentially affects liking and self-other merging

Experiment 2 revealed that liking ratings were also modulated by temporal feature-sharedness, but showed a somewhat divergent trend from Experiment 1 IOS ratings. The markedly superior predictive advantage of 1 : 1 coordination did not affect aesthetic appraisal as strongly as IOS, as illustrated by the non-significant differences between liking ratings of the three simple rhythms. Nonetheless, simple rhythms received higher overall liking ratings (by approximately 40 points) compared with the 2 : 3 and 3 : 4 polyrhythms. Furthermore, the highly complex 4 : 5 polyrhythm was not liked significantly more than the irregular rhythms, but the 3 : 4 and 2 : 3 rhythms were judged more likeable than the irregular rhythms (by approximately 75−85 points). These findings are consistent with our *a priori* hypothesis that the previously observed linear decrease in IOS for ratios except 1 : 1 was specifically modulated by the social nature of the rhythmic variation. Finally, the flat profile of liking for simple rhythms and the starkly contrasting linear decrease in liking for polyrhythms is consistent with our hypothesis that aesthetic preferences were based on the overall metrical cohesion of the rhythmic gestalt, despite the presumed sensory segregation between the two streams due to differences in sound, location and individual ‘agents’.

The distinct liking trends found for the simple rhythms and polyrhythms are at odds with earlier studies of rhythmic complexity. An inverted U-shaped ‘Wundt curve’ [98] has been found across multiple modalities in the psychology of aesthetics. The curve describes the general trend that moderately complex stimuli tend to be more aesthetically pleasing than simpler and highly complex stimuli. In music, this is postulated to be due to the pleasure derived from completing the perceptual challenge posed by somewhat complex stimuli [37,99]. Predictive coding theory offers an explanation for the curve resulting from prediction error interacting with prediction certainty in the brain. This specifically holds true for syncopation [45–48,100,101], where prediction error increases and prediction certainty decreases proportionally with increasing complexity, with the resulting certainty-weighted prediction errors mirroring the Wundt curve [46]. Given the prevalence of this phenomenon, it is interesting that our results fail to echo it between rhythm types, between levels of complexity within rhythm types, or their interaction. Instead, simple decreases in ratings with complexity, somewhat resembling patterns expected from rhythm production [102], were observed. This suggests that, despite the observational nature of the task, a purely perceptual feature-based explanation is not sufficient to explain our data. For simple rhythms, the sharp drop in appeal of the 1 : 1 rhythm down to the average liking levels of the 1 : 2 and 1 : 3 rhythms suggests that aesthetic ratings for the latter two were modulated mainly by their common feature of a relatively harmonious metrical hierarchy. This suggests relatively consistent certainty-weighted prediction errors across these metrically congruent rhythms. However, separation of the rhythmic gestalt onto two behaving agents seems to have become particularly pertinent for the metrically incongruent polyrhythms. Stated differently, the challenge of integrating multiple streams seems to have compelled participants to attend to the apparent social segregation in the stimuli, to the detriment of ratings for the more complex rhythms. Thus, similar to Experiment 1, it is plausible that the steep decreasing linear trend of liking polyrhythms may also be due to difficulty in tracking and integrating socially individuated rhythmic streams.

### 5.3. Perceptual and social mechanisms reconciling interpersonal rhythmic complexity with perfect synchrony

Given the substantial evidence that synchrony leads to prosociality, why do people choose to produce complex period-based interpersonal rhythmic variation? Synchrony often emerges coincidentally. For instance, people walking together often inadvertently synchronize their gait. But producing behaviours more rhythmically complex is seldom unintentional, and requires deliberate effort and attention towards others. Judgements of social connectedness are also sensitive to these attentional demands [11]. In extension, complex period-based coordination could be a means for signalling intentional commitment to a shared goal [103]. Therefore, individuals producing complex interpersonal rhythms could be identified as a part of the in-group due to the temporal relatedness of their actions to others’. Self-other merging judgements provided by our study offer preliminary supporting evidence for this idea. Future studies could possibly delve deeper into group-level mechanisms developing an implicit understanding of shared intentionality, interdependence, trust and cooperation through interpersonal rhythmic variation.

At a perceptual level, period-based interpersonal rhythms are inherently challenging due to their complex metrical hierarchy [104,105]. Multiple valid metrical interpretations create tension in polyrhythms [106]. Due to strong auditory-motor coupling in the brain, tension created through sound is resolved by increasing sensorimotor predictions [38]. By extension, simultaneous tracking of the segregated streams and the integrated rhythmic pattern is an embodied exercise, requiring multiple sensorimotor predictive models operating at multiple rates [107–109]. Depending on the acoustic properties (e.g. pitch, timbre and tempo) of individual streams, these model predictions can potentially be voluntarily accessed using attention-allocation strategies [110,111]. Despite the high predictive load, perceptual segregation of self-other contributions is advantageous to interpersonal rhythm production, as found in a recent study [112]. Consistent with the *auditory separation-visual integration* hypothesis, performance was overall superior when dyads made identical movements but produced different (as opposed to the same) melodies. This was attributed to auditory separation aiding self-other distinction, allowing clearer and more precise tracking of self-other actions, and simpler movement ratios enabling visuomotor entrainment. Our findings agree with this proposal, given that mechanisms underlying imagining oneself performing rhythmic coordination overlap significantly with truly performing it [39,60]. Accordingly, visuomotor entrainment based on temporal feature-sharedness could be a mechanism potentially underlying self-other merging.

Ultimately, meter may have evolved to provide a framework uniquely able to foster temporally flexible group-bonding activities that are nonetheless synchronized [30,31,113]. There are several supporting hints in this direction from the existing literature. First, *ad libitum* coordination can be highly prosocial [26], but it is unlikely to generalize across larger group sizes due to the lack of an underlying temporal architecture upon which to entrain individual movements. Meter removes this hurdle. Second, language areas in the brain, now understood to process more generally complex stimuli, are active during the perception and production of polyrhythms [114,115]. Certain jazz traditions explicitly employ polyrhythms in a manner akin to verbal communication [106,116]. Performers can also switch from easier meters to more complex meters, actively boosting audience engagement by breaking metrical expectations [106], and resulting in an amplified aesthetic experience upon the eventual resolution to a simpler meter [98]. Thus, complex metrical (polyrhythmic and possibly simpler ratio-based) hierarchies could conceivably possess a structure intrinsically conducive to aesthetic experience. Finally, musicians often invest time and effort into expanding the boundaries of rhythmic landscapes accessible to them. Polyrhythmic improvisation represents a pinnacle of such rhythmic skill. The rewarding effects of overcoming cognitive and motor challenges helps to explain musicians’ motivation to achieve such skill levels. However, another compelling aspect unique to polyrhythmic coordination may be to establish and strengthen self-other representations at an intersubjective level in the metrical hierarchy. Therefore, exploring feelings of social bondedness through complex period-based interpersonal coordination remains a fruitful avenue for future research.

## 6. Conclusions

We showed in an online perspective-taking paradigm that the extent to which temporal features are shared along a metrical hierarchy between interpersonal rhythms systematically affects feelings of self-other merging. Notably, the study highlights that self-other merging is stronger for relatively novel and complex interpersonal polyrhythms than for entirely irregular interpersonal rhythms. A second experiment showed that liking ratings for the same rhythmic variation reveal a distinct pattern from self-other merging ratings—self-other merging seems to be more biased towards 1 : 1 synchrony and decreases consistently with increasing complexity for other rhythmic ratios, while liking was preserved to a greater extent with complexity. This suggests that self-other merging in our study was specifically affected by the interpersonal nature of the rhythmic variation.

Furthermore, we demonstrate differing liking trends for two types of period-based rhythmic relationships: a flat trend for simple rhythms that share temporal features at the bar and subdivision levels, as opposed to a steep linear decreasing trend for polyrhythms that share temporal features only at the bar level. For simple rhythms, we argue that this may be attributed to aesthetic appraisal being modulated by their cohesive metrical structure. For polyrhythms, we propose that aesthetic appraisal could rely not only on high-level perceptual features but also on interpersonal features, albeit to a far lesser extent than feelings of self-other merging. Finally, we propose that the production of complex interpersonal rhythmic variation may be subserved by mechanisms of prosocial behaviour inferred through shared intentionality, and precise sensorimotor predictions aided by auditory segregation and visuomotor entrainment. Overall, our data are consistent with the hypothesis that multiple temporal aspects of joint human rhythmic behaviour play an important role in judgements of social affiliation. To the extent that our experimental findings, based on video simulations of interacting dyads, generalize to other more realistic settings, they hold promise for prospective real-world applications. Future studies could explore multimodal aspects of interpersonal rhythmic interactions in paradigms with virtual adaptive partners, and eventually extend the approach to more naturalistic dyadic and group contexts.

## Data Availability

The datasets supporting this article have been uploaded as part of the Supplementary Material [117].
